# Combination of triheptanoin with the ketogenic diet in Glucose transporter type 1 deficiency (G1D)

**DOI:** 10.1038/s41598-023-36001-x

**Published:** 2023-06-02

**Authors:** Adrian Avila, Ignacio Málaga, Deepa Sirsi, Saima Kayani, Sharon Primeaux, Gauri A. Kathote, Vikram Jakkamsetti, Raja Reddy Kallem, William C. Putnam, Jason Y. Park, Shlomo Shinnar, Juan M. Pascual

**Affiliations:** 1 Rare Brain Disorders Program, The University of Texas Southwestern Medical Center, 5323 Harry Hines Blvd., Mail Code 8813, Dallas, TX 75390 USA; 2 Department of Neurology, The University of Texas Southwestern Medical Center, Dallas, TX 75390 USA; 3 Department of Pediatrics, The University of Texas Southwestern Medical Center, Dallas, TX 75390 USA; 4 Department of Pharmacy Practice and Clinical Pharmacology, Experimental Therapeutics Center, Texas Tech University Health Sciences Center, Dallas, TX 75235 USA; 5 Department of Pharmaceutical Science, Texas Tech University Health Sciences Center, Dallas, TX 75235 USA; 6 Department of Pathology, The University of Texas Southwestern Medical Center, Dallas, TX 75390 USA; 7 Departments of Neurology and Pediatrics, Albert Einstein College of Medicine, Bronx, NY 10467 USA; 8 Department of Physiology, The University of Texas Southwestern Medical Center, Dallas, TX 75390 USA; 9 Eugene McDermott Center for Human Growth & Development/Center for Human Genetics, The University of Texas Southwestern Medical Center, Dallas, TX 75390 USA

**Keywords:** Drug development, Neurological disorders

## Abstract

Fuel influx and metabolism replenish carbon lost during normal neural activity. Ketogenic diets studied in epilepsy, dementia and other disorders do not sustain such replenishment because their ketone body derivatives contain four carbon atoms and are thus devoid of this anaplerotic or net carbon donor capacity. Yet, in these diseases carbon depletion is often inferred from cerebral fluorodeoxyglucose-positron emission tomography. Further, ketogenic diets may prove incompletely therapeutic. These deficiencies provide the motivation for complementation with anaplerotic fuel. However, there are few anaplerotic precursors consumable in clinically sufficient quantities besides those that supply glucose. Five-carbon ketones, stemming from metabolism of the food supplement triheptanoin, are anaplerotic. Triheptanoin can favorably affect Glucose transporter type 1 deficiency (G1D), a carbon-deficiency encephalopathy. However, the triheptanoin constituent heptanoate can compete with ketogenic diet-derived octanoate for metabolism in animals. It can also fuel neoglucogenesis, thus preempting ketosis. These uncertainties can be further accentuated by individual variability in ketogenesis. Therefore, human investigation is essential. Consequently, we examined the compatibility of triheptanoin at maximum tolerable dose with the ketogenic diet in 10 G1D individuals using clinical and electroencephalographic analyses, glycemia, and four- and five-carbon ketosis. 4 of 8 of subjects with pre-triheptanoin beta-hydroxybutyrate levels greater than 2 mM demonstrated a significant reduction in ketosis after triheptanoin. Changes in this and the other measures allowed us to deem the two treatments compatible in the same number of individuals, or 50% of persons in significant beta-hydroxybutyrate ketosis. These results inform the development of individualized anaplerotic modifications to the ketogenic diet.

**ClinicalTrials.gov registration** NCT03301532, first registration: 04/10/2017.

## Introduction

Evidence of reduced brain fuel entry or carbon depletion has long accompanied or characterized neurological disorders such as epilepsy^1^, dementia^2^ or trauma^3^. In man, this can be indirectly inferred from fluorodeoxyglucose-positron emission tomography (PET) or measured via microdialysis of brain tissue. In these disorders, ketogenic diets containing a large proportion of lipids relative to other nutrients are either used as therapy or constitute the subject of clinical investigation^4^. Although it is unlikely that this scientific and medical interest will ultimately equate with universal efficacy, the initial chance discovery of the effect of fasting ketosis on epilepsy^5^ has been gradually complemented with the characterization of biochemical mechanisms. As a result, today the best understood value of a ketogenic diet is the provision of alternative substrate capable of fueling the tricarboxylic acid (TCA) cycle when glucose utilization is depressed^6^.

The principal diet-derived ketone bodies, beta-hydroxybutyrate and acetoacetate, contain 4 carbons and, when metabolized, yield preferentially two molecules of two-carbon acetyl coenzyme A. This dicarbon molecule is also the main glucose oxidation byproduct. Thus, some reactions of glucose metabolism can be replaced by ketone body metabolism from the perspective of acetyl coenzyme A generation and its subsequent flux into the TCA cycle. However, a significant fraction of total brain glycolytic flux, perhaps amounting to 20%, is separately steered into anaplerosis, which is the replenishment of carbon lost in the course of the TCA cycle^7^. This carbon lost to metabolism ultimately finds its way into excretion byproducts or expired CO_2_ gas. Most available anaplerotic flux estimates refer to normal brain and their precise values vary depending on investigational methods. Yet, the importance of anaplerosis in the brain is made apparent by reductions in the activity of the enzyme that catalyzes the conversion of pyruvate into oxaloacetate, a key anaplerotic reaction. Individuals with deficiency in this enzyme, named pyruvate carboxylase, can manifest encephalopathy with necrosis of neural tissue^8^.

Considered from this perspective, the ketogenic diet is metabolically deficient: the alternative fuels provided by ketone bodies, whether ingested as conjugates of other substances or produced after ketogenic diet consumption, lack anaplerotic potential^7^. This is because dietary fats and their derivative ketone bodies contain an even number of carbons and are fully consumed in the TCA cycle via acetyl coenzyme A formation. In contrast, metabolic substrates containing an odd number of carbons greater than 5 can similarly fuel the TCA cycle through the sequential formation of one or more two-carbon acetyl coenzyme A molecules while also fueling anaplerosis via the additional generation of 3-carbon propionate as the end product of the final 3 carbons of the odd carbon substrate. Propionyl coenzyme A metabolism may then lead to the generation of the TCA cycle intermediate succinate. These reactions result in the supply of net carbon to the TCA cycle, thus compensating for a significant fraction of the carbon loss^7^.

Therefore, the ketogenic diet can be complemented to mitigate its metabolic insufficiency. To this end, we have studied individuals with partial deficiency of the brain glucose transporter type I (G1D) since they are rapidly and informatively susceptible to dietary metabolic fuel administration^9,10^. G1D is a prototypic brain carbon depletion state^11^ associated with synaptic failure that proves only partially treatable with a ketogenic diet^10^. It commonly adopts the form of a childhood-onset epilepsy refractory to antiseizure drugs which has remained almost inextricably linked to the ketogenic diet therapy for 30 years^12,13^. In this context, as in other epilepsies, dementia or trauma, which are also characterized by decreased glucose metabolism, the carbohydrate-restricted ketogenic diet leads to an intended but intuitively counterproductive decrease in blood glucose available to the brain. This is because increased glycemia interferes with ketogenesis and reduced glucose favors it.

Thus, our goal was to investigate whether triheptanoin (C7), an edible triglyceride of 7-carbon heptanoic acid, was compatible with a ketogenic diet using G1D as model disorder. Two main potential limitations to such a combination treatment exist. First, some ketogenic diets contain medium chain triglycerides, which yield the medium chain fatty acids octanoate and decanoate, both of which can compete with C7 metabolism^14^. Second, since heptanoate metabolism generates acetyl coenzyme A, C7 can potentially stimulate hepatic neoglucogenesis and this could decrease ketosis via insulin release^15^. Neoglucogenesis stemming from heptanoate has been observed after infusion of heptanoate in G1D mice^16^. However, the relative amount of heptanoate infused was greater than that derived from the C7 used for G1D subjects, since a large quantity of labeled substrate is necessary to achieve labeling of brain intermediary metabolites in human and mouse ^13^C metabolic tracer studies^17–20^. Nevertheless, uncertainty remains about the magnitude of neoglucogenesis and its potential induction of increased glycemia after C7 ingestion. Both octanoate interference and neoglucogenesis are rapid events occurring within minutes^14,16^. This informed the duration of our study of compatibility.

We used C7 at the maximum tolerable dose^21^ which, to our knowledge, has not yet been used in G1D. This dose is considerably higher than previously used^9,22^. This elevated dose is important not only to maximize any potential benefit in future studies but also to facilitate eliciting any metabolic interference with the ketogenic diet. The ultimate objective was thus to enable future combined or comparative studies because, for one third of G1D patients, the ketogenic diet is insufficient and may thus be partly replaced by C7; conversely, C7 as monotherapy may also prove insufficient in some patients and may thus benefit from the addition of a ketogenic diet^13^.

Notably, this was not a population or analytical measure distribution study, for addressing those aspects would require a different approach and a sample size unavailable for a relatively infrequent disease. Rather, the goal was to ascertain which one of three possible compatibility scenarios was more likely: (a) noninterference between C7 and the ketogenic diet, where neither the biochemical competition nor the individual metabolic variability reported in other organisms or studies precluded full compatibility, (b) generalized or absolute incompatibility due to biochemical interference, whereby any subject variability, if present, would prove trivial or insufficient to surmount the cited potentially prohibitive biochemical interactions, or (c) compatibility in only a fraction of subjects due to individual variability in one or both kinds of factors. The implication of c is that compatibility is an individual phenomenon and thus future studies or treatments must account for this crucial source of variability. This is what we found.

## Methods

### Consent, overall approach and measures of compatibility

We followed the Declaration of Helsinki of 1975 criteria as revised in 1983 and received Institutional Review Board approval from the University of Texas Southwestern Medical Center, with ClinicalTrials.gov identifier NCT03301532, first registration 04/10/2017. The inclusion and exclusion criteria are listed in Table 1. Written informed consent was obtained from one participant who was over the age of 18. Written informed consent was obtained from all the subjects or legally authorized representatives. Assent was also documented for cognitively-capable children between 10 and 17 years of age.

**Table 1:** G1D subject eligibility.

Inclusion criteria
Ages between 30 months and 35 years 11 months old, inclusive
Confirmed diagnosis of G1D genetically
Stable consumption of a ketogenic diet at 2.5:1 to 4:1 ratio (i.e., no changes in ketogenic ratio may have taken place in the 2 months prior to enrolment)
Exclusion criteria
Unrelated metabolic or genetic disease
Chronic gastrointestinal disorder, such as irritable bowel syndrome, Crohn's disease, or colitis
BMI greater than or equal to 30
Women who were pregnant or breastfeeding or planning to become pregnant during the study
Allergy or sensitivity to triheptanoin
Consumption of triheptanoin in the previous month
Consumption of medium chain triglycerides in the previous 24 h
Dementia or other progressive brain disorder
Active drug or alcohol use
Inability or unwillingness of subject or legal guardian to provide written informed consent, or assent for children age 10–17
Addition of a new antiseizure drug in the previous 3 months

The approach included substituting a fraction of ketogenic diet fat with C7, weight by weight, at the maximum tolerable dose (45% of total daily calories^21^) in individuals receiving a ketogenic diet prior to enrolment as medically prescribed independently of this study. C7 addition and equivalent dietary fat subtraction was calculated to preserve the pre-enrolment fat to protein and carbohydrate ratio. The substitution with C7 was immediate rather than gradual since patients consuming a ketogenic diet are high-fat tolerant and receive well over 45% calories from fat (often as much as 90%), such that C7 replacement was expected to be fully tolerable. To minimize gastrointestinal intolerance from triglyceride consumption, any medium chain triglyceride consumption part of the ketogenic diet was replaced with other dietary fat 24 h before triheptanoin consumption.

Because compatibility can be defined from biochemical, clinical, electroencephalographic or other perspectives, several measures of compatibility were assessed. Additionally, the data are provided or available in full to enable other possible types of compatibility analyses. First, we utilized the consideration that C7 metabolism can potentially interfere with ketosis as the key factor underlying compatibility. Thus, we used blood beta hydroxybutyric level as the primary criteria of compatibility. Second, because G1D individuals exhibit seizures, which often constitute the motivation for the use of the ketogenic diet, an expected outcome, if the ketogenic diet and C7 were compatible, was a lack of change in clinical seizures after C7. Of note, G1D seizures are not precisely quantifiable in EEG recordings lasting even a few days^10^. Since most G1D seizures are of the absence type, and because absence seizure frequency is often variable on a day-to-day basis and may remain unnoticed by patients^10^, their frequency and severity was estimated in two complementary ways. Due to the cited electrical-clinical dissociation, these two methods were not expected to necessarily yield the same result. The first method employed the same habitual caretaker for each subject, who spent the study time next to each subject and rated seizures as essentially unchanged, minimally changed (less than 30% change in frequency or duration) or significantly changed (greater than 30% change) throughout an interval that included from at least 2 days prior to the study to the end of the study. Regarding the second method, since the first method may not correlate with electrographic seizures^10^, we also assessed compatibility based upon the lack of an increase in electroencephalographic (EEG) abnormal activity. This was achieved by a clinical epileptologist (i.e., not automated) comparing of all the clinically significant abnormalities noted in pre-treatment EEGs which, for the 10 subjects studied included slowing, spikes, polyspikes or spike-waves in 48-h recordings. Based on previous G1D data illustrating about a 30% variability for test–retest in prolonged EEG recordings, and taking into consideration time of day variations and meal times^10^, changes greater than 30% in frequency following treatment measured at sequential 2-h intervals and compared at the same time of the day were considered significant.

### Participants

The disease features of all subjects included a variable combination of intellectual disability, epilepsy, ataxia, or episodic movement disorder representative of the disease population^12^. The G1D diagnosis was ascertained via DNA sequence analysis of the Slc2a1 gene, which encodes Glut1, or of the integrity of the appropriate area of chromosome 1 where the gene is located utilizing standard clinical genetic diagnostic criteria^23^. The reference Slc2a1 transcript was NM_006516.2. Enrolment followed the order of contact made by eligible subjects. Eligible contacts far exceeded enrolment targets, thus reducing potential bias associated with the sampling of small populations. There was no consideration of geographic location (U.S. or abroad) or disease severity. Some subjects were recruited from our Rare Brain Disorders Program at UT Southwestern Medical Center. They included English and Spanish speakers. Medications, including antiseizure drugs, were not allowed to change 90 days prior to or during the study. Subject age, Slc2a1 G1D causative mutation and ketogenic diet ratio are given in Table 2. As previously noted^24^, exon 4 mutations were predominant.

**Table 2:** Subject characteristics.

Subject	Age, gender	Mutation, gene location	Ketogenic diet ratio
1	21, F	L129del: c.385_387del Exon 4	2.5:1
2	11, F	R126C: c.376C>T Exon 4	2.8:1
3	10, F	R333Q: c.998G>A Exon 8	2.5:1
4	7, F	L169del: c.505_507del Exon 4	3:1
5	7, F	G154R: c.460G>A Exon 4	3:1
6	17, M	R126H: c.377G>A Exon 4	4:1
7	11, M	R153C: c.457C>T Exon 4	3.5:1
8	5, F	I386M:1158_1162del Exon 9	3.25:1
9	6, M	1p34.2, del Chromosomal deletion comprising entire Slc2a1	2.5:1
10	10, M	R126H: c.377G>A Exon 4	3.5:1

Demographic, genetic mutation and ketogenic diet ratio characteristics of the G1D subjects studied for compatibility of C7 with the ketogenic diet, numbered by order of enrolment. Age in years. M: male, F: female. Ketogenic diet ratio: proportion of fat relative to other nutrients.

### Ketogenic diet C7 supplementation

Food grade triheptanoin (Stepan Lipid Nutrition) was consumed 4 times per day (approximately every 6 h) for one day. This time was deemed sufficient for any incompatibility to manifest given the rapidity of the biochemical processes of interest^14,16^. The C7 dose was determined at 45% of the total daily caloric intake^21^. This dose was used for replacement of ketogenic fat (weight by weight). To minimize insulin-mediated suppression of ketogenesis from other foods^14^, each C7 dose was consumed 45–60 min before meals. C7 was optionally mixed with a fat free, sugar free yogurt, pudding, or equivalent low-calorie food.

### Procedures

**Figure 1:**
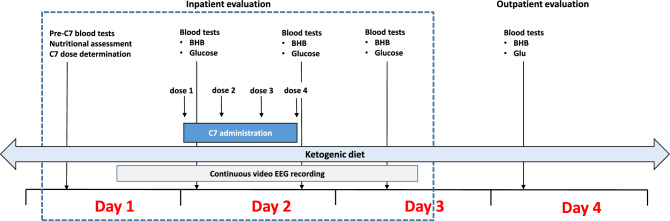
Study procedures. *BHB* beta-hydroxybutyric acid, *Glu* glucose.

Figure 1 illustrates the study procedural sequence. Each subject underwent a review of a physical exam from medical records and provided a pretreatment history including seizure frequency and received a daily physical, including neurologic, examination. The subjects were hospitalized in an Epilepsy Monitoring Unit for 48 h for C7 administration and monitoring. Continuous EEG was recorded 12 h preceding, and for the duration of C7 consumption, plus 18 h after the last dose, comprising approximately a total of 48 h. The recordings used an international 10–20 system and were reviewed and analyzed by an epileptologist. The subjects also received a nutritional assessment prior to starting the C7 supplement. Side effects were assessed using the Hague Side Effect Scale^25^ and the VA Toxicity Scale^26^.

### Laboratory tests

An analytical laboratory evaluation was obtained and reviewed on day 1, which was the day prior to starting C7. This included a comprehensive metabolic panel (glucose, blood urea nitrogen (BUN), creatinine, sodium, potassium, chloride, CO_2_, anion gap, calcium, total protein, albumin, alkaline phosphatase, aspartate aminotransferase (AST), alanine aminotransferase (ALT), total bilirubin), lipid panel, lactate, complete blood count, and beta-hydroxybutyrate. Plasma glucose and beta-hydroxybutyrate were then measured twice on days 2 and 3 approximately 2 h after the first C7 dose and 10 min after the last C7 dose on day 2. Hospital admission concluded on day 3. A complete blood count, comprehensive metabolic panel, lactate, and beta-hydroxybutyrate labs were obtained again on day 4. The subjects were also surveyed for clinical manifestations on day 5 and via telephone on day 30. Additional determinations of the C5 ketones β-hydroxypentanoate and β-ketopentanoate were performed in 5 subjects (participants 6, 7, 8, 9 and 10) at baseline and approximately 2 h approximately after the first C7 dose and 10 min after the last C7 dose on day 2, as previously described^27^.

### Estimation of beta-hydroxybutyric acid level variability in G1D under a ketogenic diet independently of C7

One method to judge C7-ketogenic diet compatibility from the perspective of beta-hydroxybutyric acid level changes relies, as prerequisite, on an estimation of beta-hydroxybutyric acid level variability in independence of C7. Clinical practice indicates that individuals who receive a ketogenic diet may exhibit significant fluctuation in blood ketone levels irrespective of the indication for the diet^28^. The same is likely to apply to G1D individuals (J.M.P. observations in n = 122 G1D subjects not treated with C7). This may impact the assessment of C7 effects on ketonemia in our C7 compatibility study subjects by introducing a source of normal variability unrelated to C7. For example, fluctuations or coefficients of variation as high as 44%^29^ and 46%^30^ (defined as the standard deviation divided by the mean of serial measurements in individual subjects) in blood beta-hydroxybutyric acid levels have been observed in individuals with epilepsy treated with a ketogenic diet.

Thus, since there were no previously reported data in G1D, a second group of subjects from our Rare Brain Disorders Program was used to estimate the variability of blood beta-hydroxybutyric acid levels in G1D subjects who consume a ketogenic diet. Thus, Institutional Review Board approval was obtained from UT Southwestern Medical Center for a retrospective chart review. This second group variability was measured in the absence of C7 consumption and was thus used as a normative or reference range for the estimation of C7 compatibility in the first group of G1D subjects. To this end, we analyzed the variation in blood beta-hydroxybutyric acid levels in 20 G1D individuals unrelated to the first group but comprising a similar age range, who were studied in our Rare Brain Disorders Program between May 2013 and November 2022. These individuals provided a total of 224 blood beta-hydroxybutyric acid values, ranging from 6 to 28 measurements per individual and spanning a minimum and maximum period of measurement of 1 month and 4 years for the individuals of this entire group. To allow for as an ample degree of variability as possible, we included subjects who consumed a ketogenic diet at the same ratio range of the subjects studied here for C7 compatibility and did not account for changes in ratio within this range for any particular individual during his or her serial measurements, nor for the time of the day when the measurements were made or time between measurements, nor for the degree of clinical therapeutic efficacy attributable to the diet. This approach was intended to provide a broad, non-experimentally controlled measure of blood beta-hydroxybutyric acid level variation consistent with fluctuations commonly observed in standard clinical practice, thus minimizing the likelihood of incorrectly attributing any observed variation after C7 to incompatibility with the ketogenic diet rather than to potentially normal variation. Because this fluctuation was measured in all cases over a significantly longer time period than the duration of our compatibility study, we reasoned that it is appropriate to use this variability as the maximum normal (i.e., C7-unrelated) variability that could be expected during our compatibility study.

To further reduce sources of C7-unrelated variability in beta-hydroxybutyric acid level comparisons pre and post C7, we also separated the analysis of the values obtained from C7-treated individuals when their level of ketosis pre-C7 treatment was below 2 mM, since any fluctuation of these values after C7 would be overshadowed by the greater degree of fluctuation estimated from the above averaged percent variability in non-C7 treated individuals. A detailed justification for this level based on the values obtained in our subjects and on other studies is discussed below.

Since future studies may determine a different degree of normal ketosis variability in G1D subjects depending on ketogenic diet ratio, time and number of blood ketone measurements or other factors, and since other numeric estimates of compatibility are also possible, we provide all of the individual values from our study to enable such future methods of analysis.

## Results

### Patient characteristics

Ten individuals with genetically confirmed G1D were enrolled to study C7-ketogenic diet compatibility (Table 2). As previously noted, Slc2a1 exon 4 mutations were common^24^. Median age at enrollment was 10 years. 6 of 10 subjects were female. Most subjects identified themselves as white (9) and non-Hispanic (9), 1 subject identified as white Hispanic and another as Asian. All of the recruited participants completed the study.

### Normative beta-hydroxybutyric acid level in G1D irrespective of C7 treatment

The mean beta-hydroxybutyric acid levels in the 20 C7-untreated G1D subjects receiving a ketogenic diet was 3.34 ± 1.86 mM (mean and SD). In these non-C7 treated subjects, the fluctuation in blood beta-hydroxybutyric acid levels was about 50%, consistent with reports in non-G1D subjects receiving a ketogenic diet^29,30^. This value also implied that, in the 10 subjects to be treated with C7, pretreatment blood beta-hydroxybutyric acid levels below 2 mM were considered as potentially too low to allow for reliable evaluation of changes relative to the larger C7-unrelated variability expected and thus this subset of subjects merited additional disaggregated analysis. A blood beta-hydroxybutyric acid level greater than 2 mM is also associated with significant ketonuria, a common clinical indicator of ketosis^29^.

### Directly observable seizures

In addition to standard Epilepsy Monitoring Unit constant and offline video supervision, all 10 subjects were almost continuously observed by a primary caretaker including at night. Parents were asked to document any apparent seizure for subsequent EEG analysis. As previously established, subjects or caretakers in general did not notice a significant fraction of electrographic seizures^10^. No subject displayed significantly increased observable seizures during the study. Two subjects exhibited complete cessation of observable seizures and one displayed a 75% reduction in seizure frequency while receiving C7. In these three subjects, seizures returned upon C7 discontinuation, as also attested by the EEG in two of them.

### EEG recordings

Of the 10 individuals the 48 h continuous video EEG was normal in 3 individuals and abnormal in 7. These results are presented in Table 3 including, for reference, previous EEG findings for each subject. As noted, the EEG remained unchanged in 7 of the 10 subjects, improved in 1 and worsened in 2, but only after discontinuation of C7.

**Table 3:** Electroencephalographic findings before and in the course of this study.

Subject	Pre-enrolment EEG		EEG immediately pre (day 1) and post (day 2) C7	
	Findings	Time before C7	Day 1	Day 2
1	Generalized spike-waves and polyspikes	7 years	Generalized spikes	Unchanged
2	Slow background	9 years	Normal	Normal
3	Slow background left hemisphere	1 year	Left intermittent temporo-parietal slowing	Reduced slowing
4	Generalized spikes, atonic and myoclonic seizures	5 years	Generalized spikes and polyspikes with partial correlation with absence seizures. Right frontal focal spikes	Normalization on C7. Upon C7 cessation, increased spike bursts, in correlation with ictal absence seizures
5	Generalized slowing. Occipital intermittent rhythmical delta activity. Multifocal spikes, left central and right frontocentral	7 months	Bilateral frontal slowing, frontocentral spike-waves. Spikes during sleep	Unchanged
6	Generalized slowing or occipital intermittent rhythmical delta activity. Bilateral frontolateral spikes Generalized polyspikes	4 months	Generalized spikes and spike-waves	Normalization on C7. Upon C7 cessation, increased spike-waves and myoclonic absence seizures
7	Generalized spike-waves up to 7 s without clinical change	6 years	Generalized or bifrontal spike-waves	Unchanged
8	Intermittently slow background, posterior parietal. Generalized epileptiform discharges	2 months	Generalized spikes upon waking	Unchanged
9	Normal	4 years	Normal	Normal
10	Normal	4 years	Normal	Normal

EEG reports prior to this study were summarized from medical records. The EEGs obtained in this study span 48 h (day 1 and day 2), comprising approximately from 12 h before C7 to 24 h after the fourth C7 dose.

### Blood analytical changes

Considering all the subjects as a group, the mean fasting blood glucose about 15 h before C7 was 81.2 ± 13.6 mg/dl (mean and SD). This value did not significantly change 30–60 min after ingestion of the first dose of C7 (79.6 ± 7.3, t test, *p* > 0.05) nor at any of the subsequent determinations (Table 4).

**Table 4:** Blood glucose values in G1D subjects in relation to C7 administration.

	Day 1 (pre C7)	Day 2 (C7)		Day 3 (post C7)		Day 4 (post C7)
Time	14:00	08:00	20:00	08:00	11:30	8:00
Subject						
1	89	83	72	98	85	85
2	82	75	85	87	103	86
3	111	77	94	87	74	89
4	86	76	88	81	87	90
5	71	78	85	86	79	77
6	77	84	87	103	98	91
7	66	64	75	90	84	87
8	66	83	88	86	83	91
9	90	91	83	92	100	93
10	74	85	86	85	83	91

Times are approximate by about ± 30 min. C7 doses were consumed on day 1 at approximately 6 a.m., 10 a.m., 2 p.m. and 6 p.m. Values are in mg/dl. ND: not determined.

**Figure 2:**
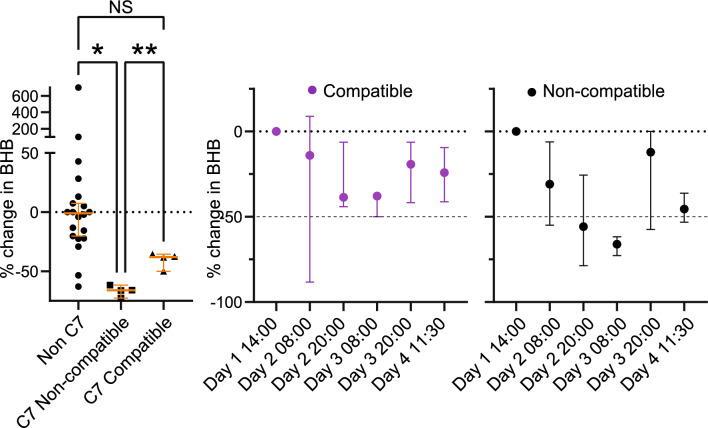
Beta-hydroxybutyrate levels independent of, before, during and after C7 ingestion. Left panel: Non C7: Percent change in 224 beta-hydroxybutyrate levels from 20 G1D individuals not receiving C7 and not treated in this study; C7 Compatible: change in G1D individuals that exhibited beta-hydroxybutyrate values indicative of compatibility with C7. C7 Non compatible: change in G1D individuals where the ketogenic diet was estimated non compatible with C7; The variability in sample-to-sample change for non-C7 treated subjects was not significantly different from the variability in G1D patients who received C7 in whom C7 was compatible with the ketogenic diet. Welch's ANOVA (for unequal variances) with Dunnett's correction for multiple comparisons. *p < 0.05 and **p < 0.01. The Browne-Forsythe test indicated similar significant differences. Right panel: Evolution of beta-hydroxybutyrate levels over time of the day for subjects where the two treatments were compatible (purple circles) or non-compatible (black circles). Compatibility from this perspective was defined as less than 50% change in beta-hydroxybutyrate levels post C7 by day 3 in relation to pre-C7 levels.

Figure 2 and Table 5 display the impact of C7 addition to the ketogenic diet on beta-hydroxybutyric acid. The impact, when present, was rapid, as expected from biochemical principles^15^ and was fully discernible by the first beta-hydroxybutyric acid level determination on day 3. Although a specific minimum level of ketosis has not been defined for the treatment of G1D, two subjects (1 and 9) displayed reduced levels pre-C7 relative to the other subjects. Based on these data, particularly on the beta-hydroxybutyric acid levels following 4 doses of C7, we estimated compatibility in subjects 3, 4, 7 and 10 and poor or no compatibility in subjects 2, 5, 6 and 8 (Fig. 2). There was no statistically discernible pattern in the two subjects (1 and 9) whose beta-hydroxybutyric acid levels were below 2 mM on day 1. There was also no obvious correlation between these levels and the subjects’ clinical manifestations and degree of treatment efficacy afforded by the ketogenic diet prior to enrolment.

**Table 5:** Ketosis, observer-noted seizures and electroencephalographic changes in G1D subjects in relation to C7 administration.

	Day 1 (pre C7)	Day 2 (C7)		Day 3 (post C7)		Day 4 (post C7)	Seizure observations	EEG abnormalities
Time	8:00	8:00	20:00	08:00	20:00	08:00		
Subject								
1	0.4	0.3	0.1	0.1	0.1	0.1	Unchanged	Unchanged
2**/***	4.7	2.6	1.0	1.6	2.0	2.2	None	None
3*	3.4	0.4	1.9	1.7	2.7	2.0	Unchanged	Reduced
4*	3.2	3.0	3.0	2.2	3.0	2.9	Ceased on C7; transient increase after C7 discontinuation	Ceased on C7; transient increase after C7 discontinuation
5**	4.7	3.9	3.5	1.8	4.7	3.0	Unchanged	Unchanged
6**	3.3	3.1	1.8	0.9	2.9	1.8	Ceased on C7; transient increase after C7 discontinuation	Ceased on C7; transient increase after C7 discontinuation
7*	6.0	4.7	3.5	3.7	3.5	4.3	Unchanged	Unchanged
8**	7.1	3.2	2.4	2.4	ND	ND	Unchanged	Unchanged
9	0.9	0.6	0.8	0.5	0.7	0.5	None	None
10*	4.5	4.9	2.9	3.1	3.7	3.6	None	None

Seizures and EEG changes are noted here in relation to the pre-C7 state (day 1). Beta-hydroxybutyrate blood levels (mM) pre and post C7. ND: not determined.

*Individuals in which the ketogenic diet was deemed compatible with C7.

**Individuals in whom the ketogenic diet was considered not compatible with C7.

***Individual for which C7 was prematurely terminated due to decreased beta-hydroxybutyrate levels. Note that subjects 1 and 9 exhibited beta-hydroxybutyrate levels below 2 mM and were thus considered uninformative from the perspective of compatibility estimation based on beta-hydroxybutyrate blood levels. The electrographic recordings are described in Table 3 in the context of all the EEGs included in this study.

Overall, beta-hydroxybutyrate levels changed from the fasting state 3.82 ± 1.96 mM to 2.67 ± 1.61 mM in the final post C7 determination on day 4. 9 of the 10 individuals presented decreased beta-hydroxybutyrate values after the first dose of C7, and all of them did so after the last dose on day 2. Over the following 48 h (days 3 and 4), beta-hydroxybutyrate increased in all individuals, but did not reach initial pre-C7 values in any of the subjects. One subject (number 2) did not receive the fourth C7 dose due to safety considerations stemming from low beta-hydroxybutyrate levels.

In summary, we found that ketosis (defined as blood beta-hydroxybutyrate level), clinical seizures, glycemia, and EEG, utilized as measures responsive to reduction of the ketogenic state, were acceptably altered by our compatibility criteria for 4 individuals supplemented with C7.

### Clinical changes including seizures and relation to EEG

There were no serious or unexpected adverse events. Seven individuals experienced mild digestive discomfort that resolved without intervention. There was no difference in frequency or severity of G1D-related symptoms or in physical examination in either C7-ketogenic diet compatible or non-compatible subjects. One subject received one dose of ondansetron after vomiting once, which had been previously used sporadically as needed for nausea and vomiting since the initiation of the ketogenic diet by this subject.

One subject improved significantly (stopped having seizures for the first time in several years) after C7 administration. Two of them exhibited worsening of seizures on day 3, after C7 discontinuation (subjects 4 and 6, Table 3). Subject 4 displayed electroclinical worsening (increased frequency of interictal paroxysms manifested as polyspike and spike and slow wave discharges as well as increased frequency of electroclinical absence seizures) 11 h after the last C7 dose, in association with a beta-hydroxybutyrate levels of 2.2 mM (from a pre-C7 level of 3.2 mM). Subject 6 also manifested electroclinical worsening approximately 11 h after the last C7 dose with increased frequency of interictal paroxysms (polyspike and spike and slow wave discharges) as well as increased frequency of electroclinical myoclonic absence seizures in relation to a beta-hydroxybutyrate level of 0.9 mM (pre-C7 level 3.3 mM).

On days 5 and 30 all appreciable clinical changes had reverted to the pre-treatment state.

### 5-carbon ketone levels

**Figure 3:**
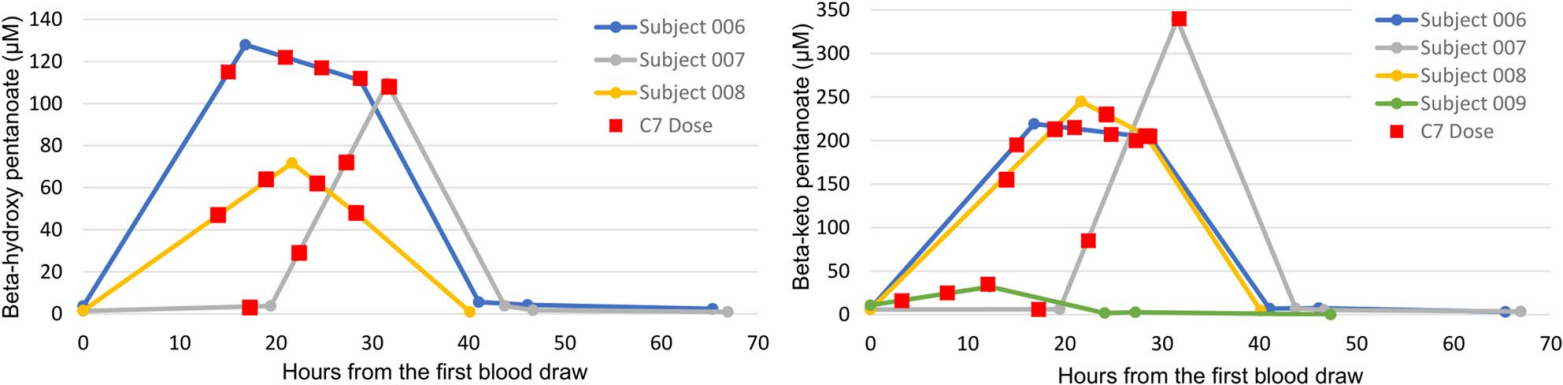
C5 ketonemia in select subjects before and after C7 administration. Left panel: Beta-hydroxy pentanoate values in relation to C7 administration times (4 doses, administered on day 2). Right panel: values for beta-keto pentanoate.

C5 ketogenesis is variable across individuals^21^. To investigate if the degree C5 ketosis influenced compatibility with the ketogenic diet, we studied several subjects serially in relation to C7 administration. Figure 3 illustrates C5 ketosis for beta-hydroxy pentanoate and beta-keto pentanoate in select subjects as a function of C7 administration. These values were commensurate with previous measurements^27^ and with determinations made at the maximum tolerable dose^21^. There was no correlation between these values and previous beta-hydroxybutyrate levels, or between these values and compatibility with the ketogenic diet.

## Discussion

Triheptanoin was previously used at a lower dose as a triglyceride food supplement to a regular human diet in individuals with Glucose transporter 1 deficiency (G1D)^9^. The goal was to replenish depleted brain carbon^31^ in a glucose-independent or complementary manner. The biochemical basis for this intervention were substantiated by multiplet ^13^C-nuclear magnetic resonance (NMR) spectroscopy^32^, gas chromatography-mass spectrometry (GC–MS) and liquid chromatography-mass spectrometry (LC–MS) studies of the metabolism of infused [5,6,7-^13^C_3_]heptanoate in a G1D mouse model faithful to the most common human disorder phenotype^16^. This metabolic substrate is primarily metabolized in the liver, producing blood [3,4,5-^13^C_3_] C5 ketones^27^. This work revealed enrichment in heptanoate-derived plasma glucose via neoglucogenesis and increased cerebral acetyl coenzyme A and glutamine concentrations. The appearance of ^13^C label in specific carbons of glutamate, glutamine and GABA was consistent with metabolism of heptanoate and or derivative C5 ketones in glia in a flux-sensitive manner^16–18,33^. Thus, the β*-*oxidation of carbons 1 to 4 of heptanoate generates two molecules of acetyl coenzyme A and one molecule of propionyl coenzyme A derived from carbons 5 to 7. The latter can enter the TCA cycle through propionyl coenzyme A carboxylase.

However, these studies, in which a regular diet was maintained, could not be directly extended to individuals who receive a ketogenic diet due to several potential limitations. First, stimulated blood ketone body levels are normally variable in persons^28^. Second, upon triheptanoin consumption, the concentrations of its two derivative C5 ketone bodies can be variable across individuals^34^ and exhibit no correlation in particular individuals^21^. Third, C5 ketone blood levels are not necessarily proportional to biological effect in the brain and therefore cannot be used alone as indicators of compatibility between triheptanoin and a ketogenic diet. This is due to the avid uptake of C5 ketones by several tissues rich in 3-oxoacid-coenzyme A transferase, including the brain^35–37^. This makes efficacy dependent upon not only blood level but also brain uptake affinity. Further, as noted in the mouse, there are several possible metabolic effects derived from heptanoate, or its byproducts, in the brain^16^. This is due to the brain fuel potential of C5 ketones, heptanoate itself, and glucose from neoglucogenesis, all of which would be difficult to mechanistically separate without co-infusion labeling or other complex studies given the uncertainties about the magnitude of some of the relevant metabolic reactions^33^.

These considerations justify our direct human investigation of compatibility from several perspectives. Previously, 14 G1D subjects on a regular diet studied in the fasting state^9^ exhibited a mean blood glucose that did not significantly change 30–60 min after a smaller C7 dose than used in this study. Beta-hydroxybutyrate levels were also unchanged in the fasting state relative to the post-C7 state. While this argued against significant neoglucogenesis from this reduced dose, the natural level of ketosis was too modest to allow inferences. The present data, obtained under the maximum tolerable C7 dose^21^, corroborate that C7 metabolism does not appreciably interfere with blood glucose level when taken simultaneously with a ketogenic diet in the time frame of this study**.**

50% or 4 of 8 subjects with initial beta-hydroxybutyrate levels greater than 2 mM demonstrated a significant reduction in ketosis after C7. This allowed us to deem C7 not compatible with the ketogenic diet in these subjects. The restoration of beta-hydroxybutyrate levels started about 30 h after the first C7 dose. However, ketone levels may not be the most valuable measure for add-on triheptanoin tolerability estimation since other factors stemming from the addition of C7 may compensate for any decrease in beta-hydroxybutyrate level.

4 of the subject families expressed overall satisfaction with symptom amelioration after C7. In all of them, C7 was deemed compatible with the ketogenic diet. Besides seizure cessation, these symptoms eluded precise quantification, since they involved facilitation of thought, expressive communication and limb coordination. Our study, however, did not evaluate these aspects. One individual (subject 6) experienced cessation of seizures after C7 addition in the context of blood glucose increase and beta-hydroxybutyrate level decrease, with a return of seizures after C7 discontinuation. This individual, who likely benefited from a slight increase in glycemia due to neoglucogenesis, may exemplify the therapeutic value of modest glucose elevations in G1D^10^. Rather than a case of incompatibility, this may be considered an instance of therapeutic substitution. Two other individuals experienced either a 75% reduction (subject 3) and cessation (subject 4) of EEG abnormalities following consumption of C7, which suggests that addition of C7 may almost immediately augment the therapeutic effect of a ketogenic diet, thus warranting further investigation.

## Conclusions

This study justifies expanding the study of triheptanoin in two contexts. First, there is a need for an alternative or a supplement to the commonly-used ketogenic diet^38^, not only to facilitate tolerability^39–42^ but also to fulfill biosynthetic demands or anaplerosis, which is deficient in numerous disease states^43,44^. Second, G1D is most symptomatic in early childhood, when brain growth parallels a robust stimulation of cerebral glucose and protein metabolism, which rely on net carbon deposition or, in short, anabolism and anaplerosis^45^. This underscores the importance of a carbon donor-rich diet, whereas the ketogenic diet is restricted in this regard. Relatively unbiased tools such a whole-exome DNA sequencing, comprehensive genomic hybridization and Sanger gene panels^23^ are increasingly uncovering G1D in young infants, for whom the ketogenic diet remains insufficiently tested^46^ and poor in anaplerotic potential during this period of rapid brain growth^47^.

## Limitations

We did not study long-term compatibility between C7 and the ketogenic diet, which may be potentially influenced by adaptation or other poorly understood processes. These hypothetical mechanisms stand in contrast with the relatively short time scale (minutes to hours) of the known relevant biochemical processes^14,16^. Therefore, the shorter time frame was selected for our investigation. Second, it is possible to define compatibility based on criteria different from ours. For example, clinical changes alone, more prolonged EEG recordings or alternative forms of data analysis such as weighted combinations of biochemical and clinical effects. Lastly, there are distinct metabolic processes for different routes and forms of administration of C7 and ketone bodies^14^. Thus, compatibility may differ under these other conditions.

## Data Availability

All the data are publicly available from the corresponding author.

## Abbreviations

C7: Triheptanoin
G1D: Glut1 deficiency
Glut1: Glucose transporter protein type 1
PET: Fluorodeoxyglucose-positron emission tomography
